# A conserved ATG2 binding site in WIPI4 and yeast Hsv2 is disrupted by mutations causing β-propeller protein-associated neurodegeneration

**DOI:** 10.1093/hmg/ddab225

**Published:** 2021-08-09

**Authors:** Miranda Bueno-Arribas, Irene Blanca, Celia Cruz-Cuevas, Ricardo Escalante, María-Angeles Navas, Olivier Vincent

**Affiliations:** Instituto de Investigaciones Biomédicas CSIC-UAM, 28029 Madrid, Spain; Departamento de Bioquímica y Biología Molecular, Facultad de Medicina, Universidad Complutense de Madrid, Madrid, Spain; Instituto de Investigaciones Biomédicas CSIC-UAM, 28029 Madrid, Spain; Instituto de Investigaciones Biomédicas CSIC-UAM, 28029 Madrid, Spain; Departamento de Bioquímica y Biología Molecular, Facultad de Medicina, Universidad Complutense de Madrid, Madrid, Spain; Instituto de Investigaciones Biomédicas CSIC-UAM, 28029 Madrid, Spain

## Abstract

PROPPINs are phosphoinositide-binding β-propeller proteins that mediate membrane recruitment of other proteins and are involved in different membrane remodeling processes. The main role of PROPPINs is their function in autophagy, where they act at different steps in phagophore formation. The human PROPPIN WIPI4 (*WDR45*) forms a complex with ATG2 involved in phagophore elongation, and mutations in this gene cause β-propeller protein-associated neurodegeneration (BPAN). The yeast functional counterpart of WIPI4 is Atg18, although its closest sequence homolog is another member of the PROPPIN family, Hsv2, whose function remains largely undefined. Here, we provide evidence that Hsv2, like WIPI4 and Atg18, interacts with Atg2. We show that Hsv2 and a pool of Atg2 colocalize on endosomes under basal conditions and at the pre-autophagosomal structure (PAS) upon autophagy induction. We further show that Hsv2 drives the recruitment of Atg2 to endosomes while Atg2 mediates Hsv2 recruitment to the PAS. *HSV2* overexpression results in mis-sorting and secretion of carboxypeptidase CPY, suggesting that the endosomal function of this protein is related to the endosome-to-Golgi recycling pathway. Furthermore, we show that the Atg2 binding site is conserved in Hsv2 and WIPI4 but not in Atg18. Notably, two WIPI4 residues involved in ATG2 binding are mutated in patients with BPAN, and there is a correlation between the inhibitory effect of these mutations on ATG2 binding and the severity of the disease.

## Introduction

Members of the beta-propellers that bind polyphosphoinositides (PROPPIN) family are seven-bladed β-propeller proteins that bind phosphatidylinositol-3-phosphate (PtdIns3P), and 3,5-bisphosphate (PtdIns(3,5)P2) and control membrane dynamics by contributing to the recruitment of additional proteins or protein complexes (1). Genetic studies in yeast identified three PROPPINs, Atg18, Atg21 and Hsv2 (YGR223C), which localize to endosomal, vacuolar and autophagic membranes (2–9). These proteins play diverse roles in autophagy, and Atg18 is also involved in the regulation of vacuolar homeostasis (2–11). In mammals, the PROPPIN family consists of four members, WIPI1–4, which are also involved in autophagy (12–15). Mutations in the WIPI genes are associated with various neurodegenerative diseases. Mutations in *WDR45* gene encoding WIPI4 cause β-propeller protein-associated neurodegeneration (BPAN, OMIM#300894), a subtype of neurodegeneration with brain iron accumulation (NBIA5) (16,17). Mutations in *WDR45B* gene encoding WIPI3 are associated with a neurodevelopmental disorder with spastic quadriplegia, brain abnormalities and seizures (NEDSBAS, OMIM#617977) while mutations affecting WIPI2 lead to intellectual developmental disorder with short stature and variable skeletal anomalies (IDDSSA, OMIM#618453) (18,19).

Macroautophagy (hereafter autophagy) is a bulk degradation process essential for cellular nutrient homeostasis. This process is initiated with the formation of a phagophore, a double membrane structure that engulfs portions of cytoplasm. The starvation-induced ULK1(Atg1)-mediated activation of the phosphatidylinositol 3-kinase complex 1 leads to the synthesis of PtdIns3P at the site of initiation of phagophore formation. PROPPINs act as PtdIns3P effectors and are involved in different steps of the autophagy process. Interaction of WIPI2 with ATG16L1 allows the recruitment of the ATG12-ATG5-ATG16L1 lipidation complex to the phagophore membrane and the subsequent lipidation of LC3 (20,21). This interaction is conserved in yeast between Atg16 and the PROPPIN Atg21 (22,23). WIPI4 and its yeast functional counterpart Atg18 form a complex with ATG2, involved in lipid transfer between the endoplasmic reticulum and the phagophore during the elongation process (24–37). WIPI3 appears to play a redundant role with WIPI4 in phagophore elongation (15,38), and consistent with this, the autophagy defect in a WIPI3 and WIPI4 double knockout mice is more pronounced than in either single knockout (39). In addition, a recent study showed that WIPI4 and WIPI3 are also involved in autophagosome–lysosome fusion (40).

The function of the other members of this protein family, WIPI1 in mammals and Hsv2 in yeast, is not well characterized. WIPI1 localizes to autophagosomes upon autophagy induction but plays only a secondary role in autophagy, probably by assisting WIPI2 in the recruitment of the lipidation machinery (12,13,15,38). In yeast, Hsv2 does not appear to be involved in bulk autophagy, although its inactivation causes a partial defect in micronucleophagy, a type of selective autophagy (3,7).

The functional analysis of proteins involved in autophagy in the yeast model has been crucial in elucidating the function of their mammalian homologs. However, the correspondence between mammalian and yeast PROPPINs is not yet clear and the phylogenetic analysis of this protein family does not completely reflect the functional similarities between family members in different species (12,13). For example, the functional counterpart of WIPI4 in yeast is Atg18, but its closest sequence homolog is Hsv2, which does not seem to be involved in bulk autophagy.

Although WIPI4 and Hsv2 appear to play different roles, the sequence homology between these proteins predicts similar functional properties. With the aim of increasing our knowledge about WIPI4 function and the mechanisms underlying BPAN disease, we investigated possible similarities between WIPI4 and yeast Hsv2. We found that Hsv2, like WIPI4, binds to Atg2 and that Hsv2 and Atg2 localize interdependently to endosomes under basal conditions and to the pre-autophagosomal structure (PAS) upon autophagy induction. We show that two residues involved in ATG2 binding site are conserved in WIPI4 and Hsv2 but not in Atg18. These amino acid residues are substituted in patients with BPAN, and there is a correlation between the severity of the clinical phenotype and the extent to which the mutations impair ATG2 binding.

## Results

### Hsv2, the closest sequence homolog of WIPI4 in yeast, interacts with Atg2

**Figure 1:**
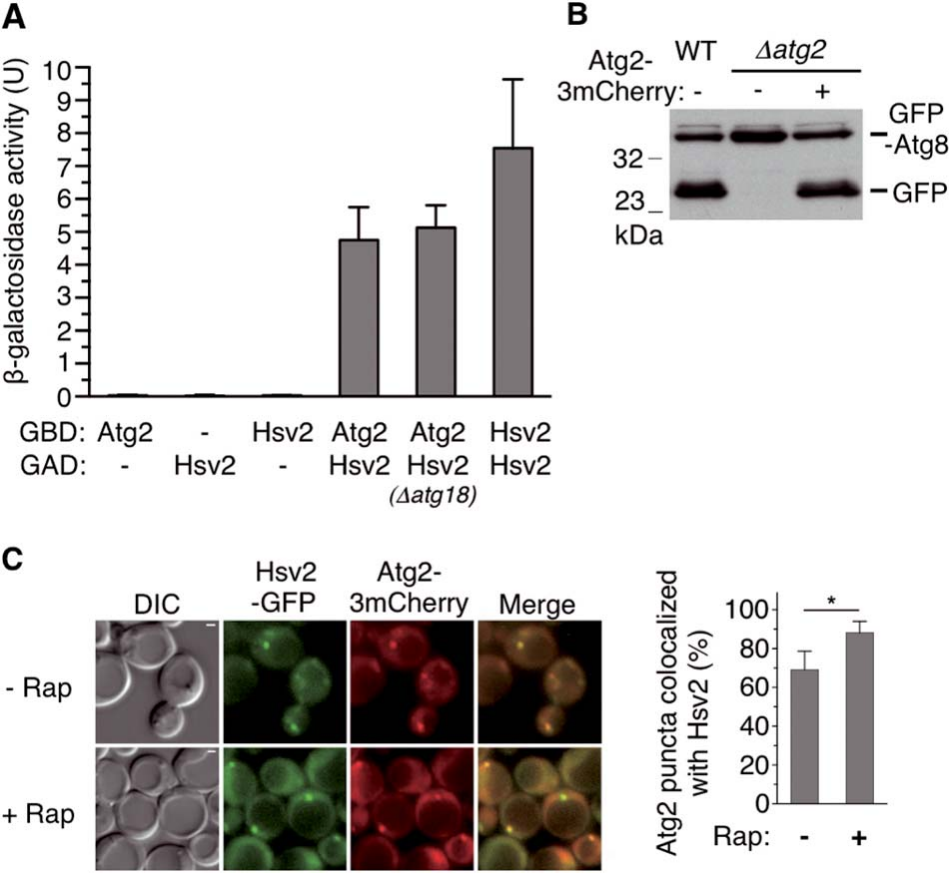
Interaction of Hsv2 with Atg2. (**A**) Two-hybrid assays. GAD-Hsv2 fusion was tested for two-hybrid interaction with GBD-Atg2 and GBD-Hsv2. Strains were Y187 or OVY546 (*Δatg18*). Values for ß-galactosidase activity are averages for eight transformants. The mean values are shown with standard deviation (SD). Values for two-hybrid control tests were <0.05 U. (**B**) Atg2-3mCherry complements the autophagic defect of a *Δatg2* mutant. Y00000 (WT) and OVY384 (*Δatg2*) were cotransformed with pGFP-Atg8 and either pAtg2-3mCherry (+) or an empty vector (−). Cells were grown to mid-log phase and starved 4 h in SD-N medium. Protein extracts were immunoblotted with anti-GFP Ab to detect GFP-Atg8 and free GFP. (**C**) Colocalization of Hsv2 and Atg2. OVY545 expressing Hsv2-GFP and Atg2-3mCherry was grown to mid-log phase (− Rap) or treated with rapamycin for 90 min (+ Rap) and examined by fluorescence and differential interference contrast (DIC) microscopy, Scale bar: 1 μm. The percentage of Atg2 puncta colocalized with Hsv2 is shown on the right. The graph represents the mean of three experiments ± SD. ^*^P < 0.05.

While previous studies indicate that the yeast functional counterpart of WIPI4 in the autophagy pathway is Atg18, its closest sequence homolog is another protein of the PROPPIN family, Hsv2 (12,13). Even though Hsv2 does not appear to be involved in bulk autophagy, the sequence similarity between WIPI4 and Hsv2 suggests that these two proteins share functional characteristics. Because WIPI4 forms a complex with ATG2, we considered the possibility that Hsv2 also interacts with Atg2 in yeast. Using the two-hybrid system, we were able to detect a strong and specific interaction between Hsv2 and Atg2 (Fig. 1A). Hsv2 also interacts with itself (Fig. 1A), suggesting that this PROPPIN can homodimerize. We also showed that Hsv2 interacts with Atg2 in a *Δatg18* mutant, thus ruling out an indirect interaction between Hsv2 and Atg2 mediated by Atg18 (Fig. 1A). To further assess the association of Hsv2 with Atg2, we tested whether these two proteins colocalize *in vivo*. We constructed a strain expressing endogenous levels of Hsv2 fused to the green fluorescent protein (GFP) and Atg2 tagged with triple-mCherry, to improve the detection of this protein. Atg2-3mCherry is functional in autophagy as it restores GFP-Atg8 processing (41) in a *Δatg2* mutant (Fig. 1B). Hsv2-GFP colocalizes with Atg2-3mCherry in discrete subcellular puncta both in growing cells and after rapamycin-mediated autophagy induction (Fig. 1C). Notably, these puncta appeared brighter in rapamycin-treated cells. Together with the two-hybrid data, these results support the idea that Hsv2, like its human homolog WIPI4 and its yeast paralog Atg18, can form a complex with Atg2.

### Atg2 mediates the recruitment of a pool of Hsv2 to the PAS upon autophagy induction

**Figure 2:**
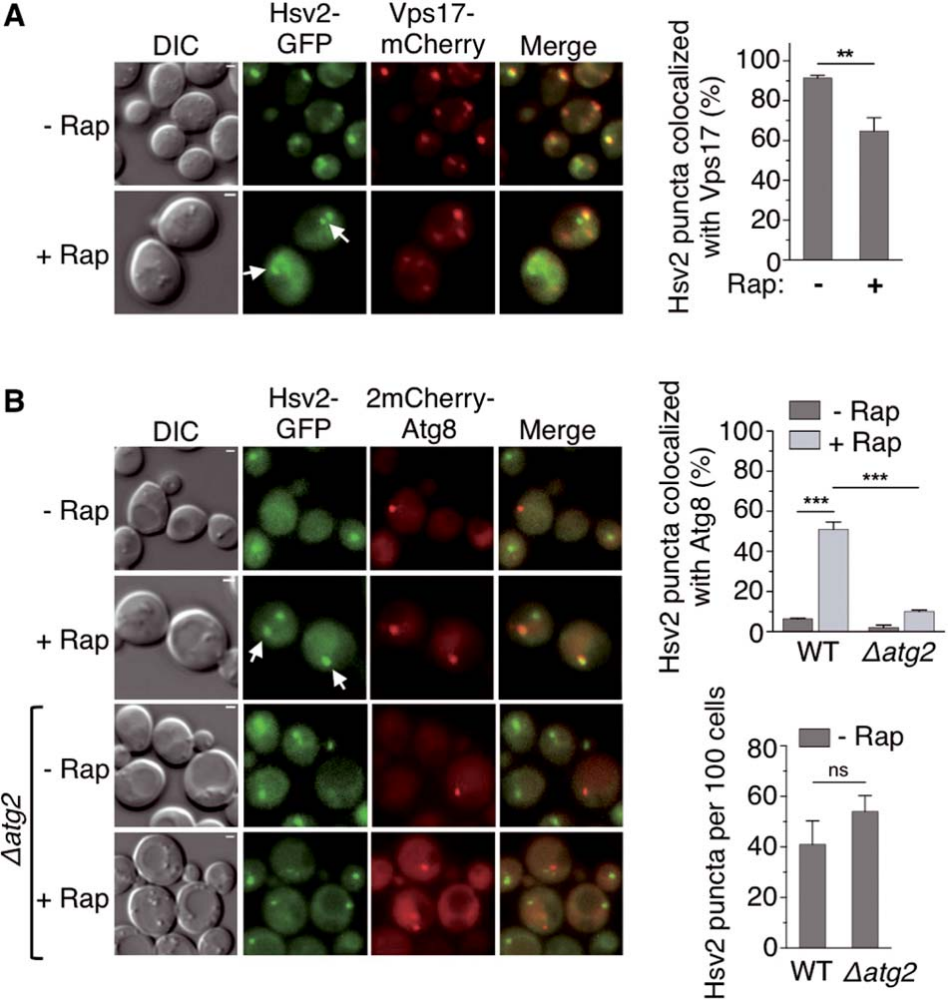
Atg2-dependent localization of Hsv2 to the PAS in autophagy-induced cells. (**A**) Colocalization of Hsv2 with the endosomal marker Vps17-mCherry. YSL829 expressing Vps17-mCherry was transformed with pHsv2-GFP. Exponentially growing (− Rap) or rapamycin-treated cells (+ Rap) were examined by fluorescence and DIC microscopy. White arrows indicate Hsv2 puncta that do not colocalize with Vps17, scale bar: 1 μm. The percentage of Hsv2 puncta colocalized with Vps17 was quantified as in Fig. 1C and is shown on the right. The graph represents the mean of three experiments ± SD. ^**^P < 0.01. (**B**) Colocalization of Hsv2 with the PAS marker 2mCherry-Atg8. OVY528 and OVY541 (*Δatg2*) expressing Hsv2-GFP were transformed with p2mCherry-Atg8 and cells were examined as above. White arrows indicate Hsv2 puncta that colocalize with Atg8. The percentage of Hsv2 puncta colocalized with Atg8 and the total number of Hsv2 puncta are shown on the right. The graph represents the mean of three experiments ± SD. ^***^P < 0.001, ns: non-significant.

The association and colocalization of Atg2 and Hsv2 were somewhat unexpected in view of the previous localization of Atg2 to the PAS and Hsv2 to endosomes (7,42–44). To resolve this issue, we decided to reinvestigate the localization of these proteins in either growing or rapamycin-treated cells. In agreement with previous work (7), Hsv2-GFP puncta colocalize with the endosomal marker Vps17-mCherry in both growing and rapamycin-treated cells (Fig. 2A). However, rapamycin-treated cells also exhibit a pool of Hsv2 in puncta that do not colocalize with Vps17 and often appear brighter (Fig. 2A). To determine whether these puncta correspond to the PAS, we tested whether Hsv2-GFP colocalizes with the PAS marker 2mCherry-Atg8. Consistent with the results obtained with Vps17-mCherry, Hsv2-GFP does not colocalize with 2mCherry-Atg8 in growing cells, whereas about half of the Hsv2 puncta localizes to the PAS upon rapamycin treatment (Fig. 2B). Remarkably, Atg2 is required for the PAS localization of Hsv2 in rapamycin-treated cells (Fig. 2B). In contrast, the number of Hsv2 puncta detected in the absence of rapamycin and corresponding to endosomes was not significantly different in a *Δatg2* mutant (Fig. 2B). Taken together, these results confirm the endosomal localization of Hsv2 but also show that Atg2 promotes the recruitment of a fraction of this protein to the PAS under autophagy-inducing conditions.

### Hsv2 mediates the recruitment of a pool of Atg2 to endosomes under basal conditions

**Figure 3:**
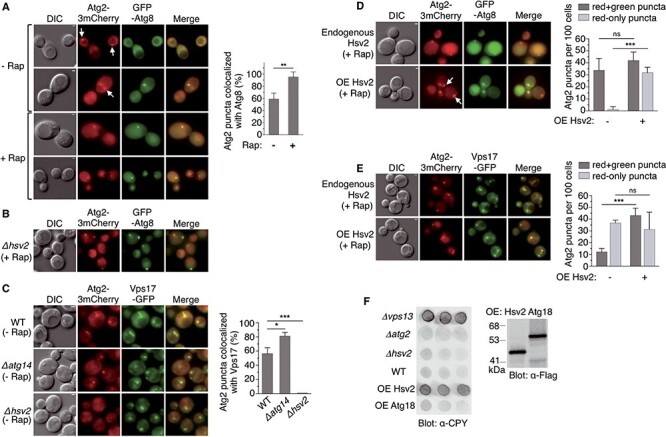
Hsv2-dependent localization of Atg2 to endosomes under basal conditions. (**A**, **B**) Colocalization of Atg2 with the PAS marker GFP-Atg8. (A) OVY384 was cotransformed with pAtg2-3mCherry and pGFP-Atg8 and examined by fluorescence and DIC microscopy in the absence or presence of rapamycin. White arrows indicate Atg2 puncta that do not colocalize with Atg8. The percentage of Atg2 puncta colocalized with Atg8 was quantified as in Fig. 1C and is shown on the right. The graph represents the mean of three experiments ± SD. ^**^P < 0.01. (B) OVY499 (*Δhsv2*) expressing Atg2-3mCherry was transformed with pGFP-Atg8. Exponentially growing cells were treated with rapamycin and examined as above. (**C**) Colocalization of Atg2 with the endosomal marker Vps17-GFP. OVY503 (WT), OVY510 (*Δatg14*) and OVY543 (*Δhsv2*) expressing Atg2-3mCherry and Vps17-GFP were grown to mid-log phase and examined as above. The percentage of Atg2 puncta colocalized with Vps17 is shown on the right. The graph represents the mean of three experiments ± SD. ^*^P < 0.05, ^***^P < 0.001. (**D**, **E**) Effect of *HSV2* overexpression (OE) on Atg2 localization in rapamycin-treated cells. (D) OVY384 was cotransformed with pAtg2-3mCherry, pGFP-Atg8 and either pADH1-Hsv2(H) (OE Hsv2) or an empty vector (endogenous Hsv2). Exponentially growing cells were treated with rapamycin and examined as above. White arrows indicate Atg2 puncta that do not colocalize with Atg8. The number of Atg2-mCherry puncta that colocalize (red + green) or not (red-only) with GFP puncta was quantified as in Fig. 1C and is shown on the right. The graph represents the mean of three experiments ± SD. ^***^P < 0.001, ns: non-significant. (E) The same experiment was performed with OVY503 expressing Atg2-3mCherry and Vps17-GFP, transformed with either pADH1-Hsv2(U) or an empty vector. (**F**) Overexpression of *HSV2* leads to CPY secretion. Y00000 was transformed with pADH1-Flag-Hsv2 (OE Hsv2), pADH1-Flag-Atg18 (OE Atg18) or an empty vector (WT), while OVY375 (*Δvps13*), OVY384 (*Δatg2*) and OVY380 (*Δhsv2*) were transformed with the empty vector. (Left) Transformants were analyzed for CPY secretion. (Right) Protein extracts from the same transformants were immunoblotted with anti-Flag Ab to detect Flag-Hsv2 and Flag-Atg18.

Similarly, we analyzed the localization of Atg2-3mCherry in growing or rapamycin-treated cells. In line with numerous studies, this protein strictly colocalizes with the PAS marker GFP-Atg8 after rapamycin-mediated autophagy induction (Fig. 3A). In contrast to the Atg2-dependent localization of Hsv2 to the PAS, *HSV2* deletion does not impair the PAS localization of Atg2 in rapamycin-treated cells (Fig. 3B), which is consistent with the proper functioning of bulk autophagy in this mutant (9). Unexpectedly, about half of the Atg2 puncta are not associated to the PAS in the absence of rapamycin (Fig. 3A). To determine whether these non-PAS puncta are endosomes, we tested whether Atg2-3mCherry colocalizes with the endosomal marker Vps17-GFP in growing cells. Consistent with the results obtained with GFP-Atg8, we observed that about half of the Atg2 puncta are associated with endosomes (Fig. 3C). In agreement with the endosomal localization of this pool of Atg2, these puncta are still detected in a *Δatg14* mutant that lacks the phosphatidylinositol 3-kinase complex 1 required for the PAS localization of Atg2 (45) (Fig. 3C). We note that the number of Atg2 puncta colocalizing with endosomes is even higher in the *Δatg14* mutant, most likely due to the availability of the Atg2 pool that no longer associates with the PAS. Remarkably, the endosomal localization of Atg2 is undetectable in a *Δhsv2* mutant (Fig. 3C). This result supports the idea that Hsv2 mediates the recruitment of a fraction of Atg2 to endosomes under basal conditions, when autophagy is not induced.

With the aim of further exploring the role of Hsv2 in the recruitment of Atg2 to endosomes, we analyzed the effect of *HSV2* overexpression on Atg2 localization in rapamycin-treated cells, wherein Atg2 localizes almost exclusively to the PAS. We found that *HSV2* overexpression results in the appearance of Atg2-3mCherry puncta that do not colocalize with the PAS marker GFP-Atg8 (Fig. 3D). In contrast, the number of Atg2 puncta corresponding to the PAS remains approximately the same, consistent with previous work showing that *HSV2* overexpression does not interfere with the autophagy pathway (7). To ascertain whether these new puncta are endosomes, we repeated this experiment with the endosomal marker Vps17-GFP. Consistent with the above data, *HSV2* overexpression leads to a marked increase in the number of Atg2-3mCherry puncta that colocalize with Vps17-GFP (Fig. 3E). Taken together, these results demonstrate that *HSV2* overexpression drives the recruitment of Atg2 to endosomes.

The localization of Hsv2 and Atg2 to endosomes suggests that these proteins may play a role in endosomal trafficking. In addition, the Atg2-related protein Vps13 also localizes to endosomes and a *Δvps13* mutant is defective in endosome-to-Golgi retrieval of Vps10, the sorting receptor for the vacuolar protease carboxypeptidase Y (CPY), resulting in CPY secretion (46) (Fig. 3F). In agreement with previous work (7), we did not detect CPY secretion in *Δhsv2* or *Δatg2* mutants (Fig. 3F). However, we found that overexpression of *HSV2*, but not of *ATG18*, results in CPY secretion to almost the same extent as *VPS13* deletion (Fig. 3F). This result suggests that the endosomal function of Hsv2, and possibly of the complex formed by Hsv2 and Atg2, might be like Vps13, related to the endosome-to-Golgi recycling pathway.

### Identification of a conserved Atg2 binding site in Hsv2 and WIPI4

**Figure 4:**
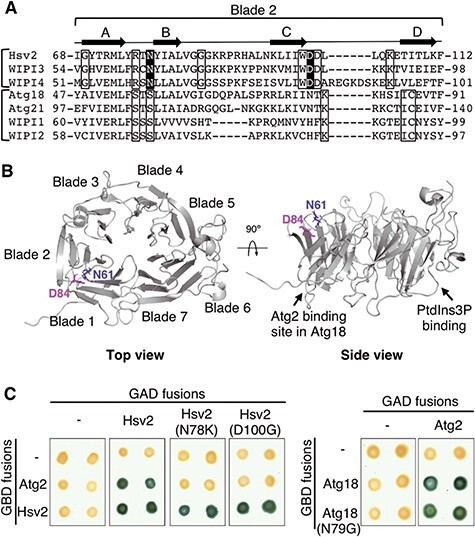
Identification of a conserved Atg2 binding site in Hsv2 and WIPI4. (**A**) Alignment (T-Coffee) of the propeller blade 2 in human (WIPI) and yeast PROPPINs. Brackets indicate the two PROPPIN subfamilies, one containing Hsv2, WIPI3 and WIPI4, and the other containing Atg18, Atg21, WIPI1 and WIPI2. Fully conserved residues specific to each subfamily are indicated by open boxes, and the two conserved residues mutated in WIPI4 in BPAN patients (N61K and D84G) are shaded in black. The four β-stranded antiparallel β-sheet (A–D) of the propeller blade 2 are shown above the sequences. (**B**) Localization of the N61K and D84G substitutions in the 3D structure of WIPI4. WIPI4 structure was predicted using Robetta (http://robetta.bakerlab.org) based on the structure of Hsv2 from *Kluyveromyces lactis* (PDB: 4AV9). Top and side views of WIPI4 structure were generated with PyMOL software (The PyMOL Molecular Graphics System, Version 2.4.0 Schrödinger, LLC). Mutated residues are shown as blue and pink sticks. The localization of the Atg2 binding site in Atg18 and the PtdIns3P binding site are marked by an arrow. (**C**) Two-hybrid assays. Left: GAD-Hsv2 fusion and the N78K and D100G mutant derivatives were tested for two-hybrid interaction with GBD-Atg2 and GBD-Hsv2. Right: The same assay was performed with GAD-Atg2 and GBD-Atg18 and the N79G mutant derivative. Positive interactions were detected by ß-galactosidase lift filter assays.

Previous studies have shown that the interaction of WIPI4 with ATG2A/B involves a region that spans blades 2 and 3 of the β-propeller (30,33). Additionally, studies of the genetic basis of BPAN disease led to the identification of two missense mutations in the *WDR45* gene causing N61K and D84G residue substitutions in the blade 2 of WIPI4 (16,47). The two mutant residues belong to the loops connecting β-strands A–B and C–D in blade 2 (Fig. 4A) and are adjacent residues in the 3D structure of the protein (Fig. 4B). These residues protrude from the top surface of the β-propeller and are on the opposite side of the PtdIns3P binding site (Fig. 4B). The location of these residues and their substitution in BPAN patients suggest that they could be involved in the interaction of WIPI4 with ATG2. In addition, these residues are fully conserved in the PROPPIN subfamily containing Hsv2, WIPI3 and WIPI4 (12,13) (Fig. 4A), which might indicate that the ATG2 binding site is conserved among these proteins. In order to test this hypothesis, we introduced the corresponding mutations (N78K and D100G) in yeast Hsv2 and analyzed their effect on Atg2 binding in two-hybrid assays. We found that both mutations result in a complete loss of the interaction with Atg2 (Fig. 4C). In contrast, they have no effect on the interaction of Hsv2 with itself (Fig. 4C), thus showing that the effect on Atg2 binding is specific and is not due to increased instability of the mutant proteins. Thus, our results indicate that these residues mediate the interaction of Hsv2 with Atg2. Interestingly, the two residues are conserved in Hsv2 and WIPI4 but not in Atg18, which also binds to Atg2 (Fig. 4A). Multiple sequence alignment indicates that one of the mutated residues in Hsv2 (D100) corresponds to the N79 residue in Atg18 (Fig. 4A). We analyzed the effect of the equivalent substitution in Atg18 (N79G) on Atg2 binding in two-hybrid assays. We found that this mutation does not affect the interaction between the two proteins (Fig. 4C). These results, together with the absence of conservation of the two residues in Atg18, suggest that the binding site for Atg2 is conserved in Hsv2 and WIPI4 but not in Atg18.

### Disease-causing mutations prevent binding of WIPI4 to ATG2A

**Figure 5:**
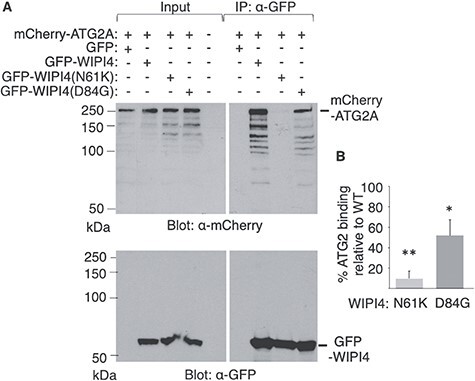
Effect of the WIPI4 substitutions N61K and D84G on ATG2A binding. Coimmunoprecipitation of ATG2A and WIPI4 from HeLa cell extracts. Cells were cotransfected with pmCherry-ATG2A and pEGFP-C3, pEGFP-WIPI4 or the indicated mutant derivatives. (**A**) Cells extracts were immunoprecipitated (IP) with *GFP-trap* beads and immunoblotted with anti-mCherry antibody (upper panel) and, after stripping, with anti-GFP antibody (lower panel). Due to its small size, unfused GFP was detected in another immunoblot (data not shown). Input indicates 10% of protein extracts used for immunoprecipitation. In the upper panel, autoradiographic exposure time was 2 h for inputs and 45 min for IP samples. A representative experiment is shown. (**B**) Densitometric analysis of the mCherry-ATG2A band was performed with ChemiDoc^TM^ Imaging System and ImageLab software and plotted as IP to input ratio for each mutant relative to the wild-type (WT) value. Data represent means ± SD of three independent experiments. *P* values were determined by unpaired two-tailed Student’s *t*-test. ^*^P < 0.05, ^**^P < 0.01.

**Figure 6:**
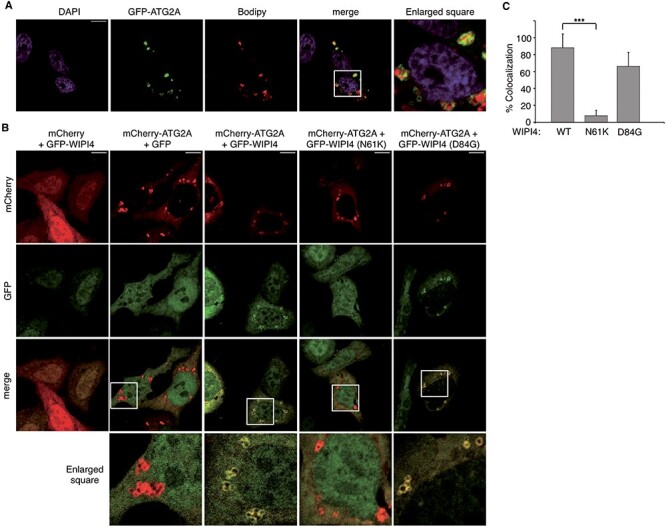
Effect of the WIPI4 substitutions N61K and D84G on the colocalization of WIPI4 and ATG2A. (**A**) Representative confocal fluorescence images of HeLa cells transfected with pGFP-ATG2A and stained for lipid droplets with Bodipy and nuclei with DAPI. (**B**) Representative confocal fluorescence images of HeLa cells cotransfected with pmCherry or pmCherry-ATG2A and pEGFP-C3, pEGFP-WIPI4 or the indicated mutant derivatives. GFP, mCherry and DAPI fluorescence channels are shown. Scale bar 10 μm. White box represent enlarged area shown. (**C**) Percentage of mCherry-ATG2A structures that colocalize with GFP-WIPI4. Data represent means ± SD of at least 283 mCherry-ATG2A positive structures in 87 co-transfected cells from five independent transfections. *P* values were determined by unpaired two-tailed Student’s *t*-test. ^***^P < 0.001.

Based on the results obtained from yeast, we analyzed the effect of the disease-causing N61K and D84G substitutions on the *in vivo* association of GFP-WIPI4 and mCherry-ATG2A fusion proteins in HeLa cells. Previous work has shown that the interaction between exogenously expressed WIPI4 and ATG2A can be detected by co-immunoprecipitation both under nutrient-rich and starvation condition (15). Consistent with this, we observed that mCherry-ATG2A co-immunoprecipitates with GFP-WIPI4 (Fig. 5A). We found that the N61K substitution abolishes the association between these two proteins while the D84G substitution causes only a 50% reduction of co-immunoprecipitated ATG2A (Fig. 5A and B). To confirm these results, we analyzed the effect of these mutations on the colocalization of GFP-WIPI4 and mCherry-ATG2A in HeLa cells. Earlier studies have shown that exogenously expressed ATG2A associates mainly with lipid droplets and to a lesser extent with autophagic structures under starvation conditions (36,48–50). In keeping with these findings, we observed that under nutrient-rich conditions, GFP-ATG2A is localized in circular structures stained with the Lipid-Droplet Marker Bodipy 558/568 C12 (Fig. 6A). In contrast, GFP-WIPI4 is diffusely localized in the cytosol with some nuclear enrichment (Fig. 6B, first column). In GFP-WIPI4/mCherry-ATG2A cotransfected cells, the two proteins colocalize on the circular structures, thus indicating that ATG2A can mediate the recruitment of WIPI4 to lipid droplets (Fig. 6B). We therefore used these conditions to monitor the effect of residue substitutions in WIPI4 on the *in vivo* interaction with ATG2A. In line with the co-immunoprecipitation data, we found that the N61K substitution abolishes the colocalization of WIPI4 with ATG2A in lipid droplets while the D84G substitution appears to have only a minor effect (Fig. 6B and C). Altogether, these results demonstrate that the Atg2 binding site is conserved in WIPI4 and Hsv2 and that the N61K substitution in WIPI4 has a much stronger effect that D84G on ATG2A binding.

## Discussion

Previous studies have shown that WIPI4 and its yeast functional counterpart Atg18 form a complex with ATG2, involved in phagophore elongation in the autophagy pathway (24–37). Here we provide evidence that yeast genome encodes a second Atg2 interactor, Hsv2, which is the closest sequence homolog of WIPI4 in this organism. We show that Hsv2 and Atg2 interact strongly and specifically in the two-hybrid system and localize interdependently to endosomes under basal conditions and to the PAS after autophagy induction. We further demonstrate that the Atg2 binding site in Hsv2 is conserved in WIPI4 and disrupted in BPAN patients.

Consistent with previous studies, Hsv2-GFP localizes mainly to endosomes, but we found that a pool of this protein localizes to the PAS under autophagy-inducing conditions. We further showed that the localization of Hsv2 to the PAS requires Atg2. The behavior of Hsv2 is therefore similar to that of Atg18, which localizes mainly to endosomes and is recruited in an Atg2-dependent manner to the PAS upon autophagy induction (25). Likewise, analysis of Atg2-3mCherry confirmed the almost exclusive localization of this protein to the PAS in autophagy-induced cells but also revealed the existence of a pool of Atg2 that localizes in an Hsv2-dependent manner to endosomes under basal conditions. These data, taken together with the colocalization of Hsv2 and Atg2 in both growing and rapamycin-treated cells, support the idea that a fraction of Hsv2 and Atg2 are associated in a protein complex, which localizes to endosomes under basal conditions and to the PAS under autophagy-inducing conditions. The endosomal localization of Atg2 requires Hsv2, and *HSV2* overexpression drives the recruitment of Atg2 to endosomes, thus indicating that Hsv2 is the main responsible for the endosomal localization of this complex. In contrast, Atg2 is required for the PAS localization of Hsv2 and not vice versa, most likely because Atg2 interacts with Atg9 at the autophagosomal membrane (27). In addition, the C-terminal region of Atg2 has been shown to bind PtdIns3P in a calcium-dependent manner (51,52). Notably, Hsv2 plays a similar endosomal adaptor role for Atg2 as the sorting nexin Ypt35 for the Atg2-related protein Vps13, as *YPT35* overexpression also drives recruitment of Vps13 to endosomes (53).

Although previous work suggests that Atg2 may localize to structures other than the PAS (51), it is surprising that the endosomal pool of this protein has not been detected previously. One possible reason is that, with the exception of initial studies (42,43), the localization of Atg2 has been most extensively studied under autophagy-inducing conditions, in which Atg2 localizes almost exclusively to the PAS. In addition, early studies have been carried out with N-terminal GFP-tagged Atg2, which is only partially functional (43). Finally, the C-terminal triple mCherry fusion greatly enhances the detection of endosomal puncta, which often appear as a less intense fluorescent signal than PAS puncta.

The endosomal and PAS localization of Hsv2 and Atg2 raises questions about the function of these proteins in these different subcellular localizations. The localization of Hsv2 to the PAS in rapamycin-treated cells was unexpected since the corresponding mutant is not defective in bulk autophagy (9). However, this lack of apparent phenotype may be due to a functional redundancy between Hsv2 and another component of the autophagy machinery, as described for another PROPPIN, Atg21, which functions redundantly with the Atg1 complex in recruiting the lipidation machinery to the PAS (54). On the other hand, Hsv2-mediated recruitment of Atg2 to the endosomal membrane could provide a reservoir of Atg2 under basal conditions, when autophagy is not induced. However, the effect of *HSV2* overexpression on CPY sorting suggests a possible involvement of this protein in endosome-to-Golgi traffic. This effect is not observed with Atg18, which also localizes to endosomes (25), and therefore is not simply due to a competition for PtdIns3P binding that could interfere with endosome dynamics. The effect of *HSV2* overexpression on CPY secretion is surprising since the same effect has been previously reported for another PROPPIN, Atg21, but not for Hsv2 (7). However, the discrepancies between the reported phenotypes could be due to the different genetic background of the strains used in the two studies. *HSV2* has not previously been identified in yeast trafficking mutant screens, but this could be due to the high level of redundancy of recycling pathways between the Golgi and endosomes. Regarding the endosomal localization of Atg2, it is striking that the Atg2-related protein Vps13 also localizes to endosomes and is required for CPY receptor recycling. The hypothetical involvement of Atg2 in Golgi/endosome trafficking is further supported by the identification of this gene as a suppressor of defects in biogenesis of lysosome-related organelles of endosomal adaptor protein-3 (AP-3) mutants in *Drosophila* (55).

Interestingly, one of the two WIPI1 isoforms in mammals, WIPI1β (WIPI49), localizes to endosomal and Golgi membranes, and as with Hsv2, overexpression of WIPI1β disrupts the proper function of the mannose-6-phosphate receptor pathway (56), which plays the same role as the CPY recycling pathway in yeast (57). Additionally, a recent study showed that WIPI1 is directly involved in the formation and fission of endosomal carriers (58). The similarities between WIPI1 and Hsv2 may indicate that these two proteins are functionally related, as are WIPI4 and Atg18, which are both involved in phagophore elongation. Intriguingly, these possible functional similarities between yeast and mammals do not reflect the phylogenetic tree that distinguishes two subfamilies, one containing WIPI4 and Hsv2 and the other containing WIPI1 and Atg18 (12,13).

Previous studies showed that the main binding site of ATG2A/B in WIPI4 is located in a region that includes blades 2 and 3 of the β-propeller (30,33). We identified two conserved residues in blade 2 of WIPI4 and Hsv2, which are adjacent in the 3D structure and mediate ATG2A/Atg2 binding, implying a conserved recognition mechanism in yeast and mammals. While this manuscript was in preparation, the structural analysis of another PROPPIN, WIPI3, in complex with a ATG2A fragment confirmed the involvement of one of these residues in binding ATG2A (59). We found that the effect of substituting one of these amino acids residues is different in yeast and mammals and that one of the WIPI4 mutations only results in a partial loss of binding. This difference may be due to the presence of nearby residues in WIPI4, which are not conserved in Hsv2 and can partially compensate the deleterious effect of the mutation. Notably, the two residues identified in WIPI4/Hsv2 are not conserved in Atg18, suggesting that the Atg2 binding site in Atg18 is different. This possibility is consistent with the identification of Atg18 mutations that prevent Atg2 binding and are located on the opposite side of the β-propeller (Fig. 4B) (26) and with the identification of an additional Atg2 binding site in blade 7 (60). This opens up the possibility of a different binding surface on Atg2 for these two proteins, which could allow simultaneous binding of the two PROPPINs to the same Atg2 molecule.

The two WIPI4 residues that mediate ATG2A binding are substituted in patients with BPAN, and we show that these two substitutions partially or completely disrupt the interaction between the two proteins. These results support the idea that BPAN is due to a defect in the function of the WIPI4-ATG2 complex. This finding is relevant for the understanding of the pathogenesis of BPAN since the study of the functional counterpart of WIPI4, Atg18, has shown that these proteins can also function independently of their association with Atg2 in other processes such as the regulation of vacuolar homeostasis in yeast (11). The function of PROPPINs in mammals is also not restricted to autophagy, as shown by the role of WIPI1β (WIPI49) in the traffic between trans-Golgi Network and endosomes (56). WIPI4 has not been previously reported to be involved in processes other than autophagy, and like Hsv2 and Atg2 in yeast, both WIPI4 and ATG2A localize at least partially to the PAS under autophagy-inducing conditions (14,15,40,48,49,61). However, ATG2A also predominantly localizes to lipid droplets and this protein has been shown to regulate the size and distribution of these organelles (48,49). Accordingly, we found that under nutrient-rich conditions, exogenously expressed mCherry-ATG2A localizes to lipid droplets. Unexpectedly, mCherry-ATG2A and GFP-WIPI4 colocalize on lipid droplets when the two proteins are co-expressed, suggesting that ATG2A could mediate the recruitment of WIPI4 to these organelles. Such localization of WIPI4 is not observed in cells expressing only the GFP-WIPI4 fusion, suggesting that endogenous levels of ATG2A are too low to produce detectable recruitment of exogenously expressed GFP-WIPI4 to these structures. In yeast, Atg2 and PtdIns3P binding promote the recruitment of Atg18 to the phagophore membrane, and here we show that Atg2 also drives the recruitment of Hsv2 to the PAS. The Hsv2-dependent localization of Atg2 to endosomes in growing cells also indicates that the localization of WIPI-ATG2 complexes in yeast is not restricted to the PAS. However, we cannot rule out the possibility that the ATG2A-dependent localization of WIPI4 to lipid droplets is due to the overexpression of these two proteins, and future studies of the endogenous proteins will be necessary to determine whether the WIPI4-ATG2A complex can also localize to these organelles, especially under nutrient-rich conditions when autophagy is not induced.

BPAN is a rare neurodegenerative disorder with a biphasic set of neurological symptoms. Patients present with global developmental delay and intellectual disabilities during childhood and develop later other symptoms such as dystonia and parkinsonism. Most affected individuals are female carriers of *de novo* heterozygous mutations in the *WDR45* (WIPI4) gene located in the X chromosome. To date, only a few missense mutations have been identified (62). Our results indicate that one of these mutations, causing N61K substitution, abolishes ATG2A binding. This mutation results in the same severe clinical phenotype as frameshift or nonsense mutations predicted to cause full loss of function (16,63), thus supporting the idea that the interaction with ATG2 is essential for the disease-related function of WIPI4. Interestingly, the D84G substitution is associated with a unique clinical phenotype characterized by mild cognitive delay (47,63). In contrast to most of BPAN children who have no expressive language, this patient had nearly normal language development. Remarkably, our results indicate that the D84G substitution leads only to a partial defect in the interaction with ATG2A. Thus, our findings strongly support the hypothesis that the unexpectedly mild phenotype associated to this mutation results from the ability of the mutant protein to interact, albeit less strongly, with ATG2. Overall our results presented here show that the functional analysis of missense *WDR45* (WIPI4) mutations identified in BPAN patients may provide new insights into the clinical phenotype of milder cases of BPAN and expand our knowledge of the molecular mechanisms underlying this disease.

## Materials and Methods

### Yeast strains and genetic methods

*Saccharomyces cerevisiae* strains used in this study are described in Supplementary Material, Table S1. PCR-based gene deletion with the hphNT1 and natMX4 markers was performed as described previously (64,65). To tag the chromosomal *VPS17* gene with GFP, we used a PCR-based gene targeting method and the plasmid pFA6a-GFP(S65T)-KanMX6 (66). The strains expressing C-terminally GFP-tagged Hsv2 and triple mCherry-tagged Atg2, under the control of their native promoter and the *ADH1* terminator, were constructed by genomic integration of pRS306 and pRS303 (67) derivatives of pHsv2-GFP and pAtg2-3mCherry, at the *URA3* and *HIS3* locus of W303-1A, respectively. Standard genetic methods were followed, and yeast cultures were grown in yeast extract-peptone-adenine-dextrose (YPAD) or synthetic dextrose (SD) medium lacking appropriate supplements when plasmid selection was required (68). Autophagy was induced by nitrogen starvation in SD-N medium for 4 h (0.17% yeast nitrogen base without amino acids and 2% glucose) or by rapamycin treatment for 90 or 60 min when 2mCherry-Atg8 was used as PAS marker.

### Plasmids

Two-hybrid plasmids encoding Gal4 binding domain (GBD) or Gal4 activation domain (GAD) fusions to Atg2, Hsv2 and Atg18 were constructed by cloning the corresponding coding sequences in the BamH1 site of pGBKT7 or pACT2 (Clontech, Palo Alto, CA, USA), respectively. pADH1-Hsv2(H) and pADH1-Hsv2(U) containing the *HSV2* ORF under the control of the *ADH1* promoter and terminator are derivatives of the multicopy plasmids pRS423 and pRS426 (67), respectively. pADH1-Flag-Hsv2 and pADH1-Flag-Atg18 were generated by cloning the Hsv2 and Atg18 coding sequences in the BamH1 site of a derivative of multicopy plasmid pSK134 containing a triple Flag epitope (69). pHsv2-GFP and pAtg2-3mCherry encoding C-terminally GFP-tagged Hsv2 and triple mCherry-tagged Atg2 under the control of their native promoter and the *ADH1* terminator are derivatives of centromeric plasmids pRS316 and pRS315 (67), containing the Hsv2 and Atg2 coding sequence with 500 bp 5′ sequence. In addition, pHsv2-GFP contains a (GGGGS)2 linker at the C-terminus of Hsv2. pGFP-Atg8 (pRS316-GFP–AUT7) and p2mCherry-Atg8, a pRS313 derivative of pRS314[2×mCherry-ATG8], have been described previously (44,70). pEGFP-WIPI4 and pmCherry-ATG2A were generated by cloning the WIPI4 and ATG2A coding sequences in the polylinker of pEGFP-C3 (Clontech) and pmCherry, a pEGFP-C3 derivative in which the GFP sequence has been replaced by mCherry. WIPI4 and ATG2A coding sequences were obtained from pCAG-WIPI4 (71) and pEGFP-C1-hAtg2A (48), which were gifts from Noboru Mizushima (Addgene plasmid # 36456; http://n2t.net/addgene:36456; RRID:Addgene_36456) and James Hurley (Addgene plasmid # 42528; http://n2t.net/addgene:42528; RRID:Addgene_42528), respectively. Single mutations in Hsv2, Atg18 and WIPI4 were introduced by site-directed mutagenesis.

### HeLa cell culture, transient transfection and fluorescence microscopy

HeLa cells were cultured in DMEM supplemented with 10% fetal bovine serum, 2 mmol/l l-glutamine, 100 U/ml penicillin/streptomycin (all Gibco/Life Technologies, Carlsbad, CA, USA) at 37°C in a 5% CO humidified atmosphere. Transient transfections were performed with lipofectAMINE 2000 (Invitrogen, Carlsbad, CA, USA) in 70% confluent culture dishes. For fluorescence microscopy, cells were cultured on 12 mm diameter coverslips (Menzel-Glaser, Madison, WI, USA) in 6-well culture dishes and cotransfected with 1 μg of pmCherry-ATG2A or pmCherry along with 1 μg of pEGFP-C3, pEGFP-WIPI4 or the indicated mutant derivatives. Twenty-four hours after transfection, cells on coverslips were fixed with 4% paraformaldehyde and coverslips were mounted with Fluoromount G (Electron Microscopy Sciences, Hatfield, PA, USA). For lipid droplet staining, cells transfected with 1 μg of pEGFP-ATG2 were incubated on phosphate-buffered saline with 1 μg/ml BODIPY 558/568-C12 (Molecular Probes, Eugene, OR, USA) for 15 min, and after fixation, nuclei were stained with 4′,6-diamidino-2-phenylindole (DAPI, Invitrogen). The analysis of fluorescent proteins was performed with a microscope confocal spectral LSM710 (Zeiss, Oberkochen, Germany) and images were captured with a Plan-APOCHROMAT 63x objective lens. Excitation wavelengths of 405, 488 and 543 nm were used for detection of DAPI, GFP and mCherry, respectively. Confocal images were processed and fluorescence intensities of images were quantified with the ImageJ 1.48v software (National Institutes of Health).

### Yeast fluorescence microscopy

Yeast cells expressing GFP- and mCherry-tagged proteins were either grown to mid-log phase in SD medium to select for plasmids, or in YPAD medium before being resuspended in fresh SD medium. GFP and mCherry localization in live cultures were visualized by fluorescence microscopy as previously described (72). The number of GFP/mCherry puncta was determined from three independent experiments and at least 100 cells were counted for each experiment. The mean values are shown with standard deviation. *P*-values were determined by unpaired two-tailed Student’s *t*-test. *P*-values <0.05 were considered statistically significant.

### Immunoblot analysis

Yeast protein extracts prepared by the NaOH/trichloroacetic lysis method (73) were analyzed by SDS/PAGE and immunoblotting with anti-GFP (G-1544; Sigma-Aldrich, St Louis, MO, USA) or anti-Flag (M2, Sigma-Aldrich). To examine the secretion of CPY, serial dilutions of yeast culture were spotted on a nitrocellulose filter placed over a SD plate. After incubation at 30°C for 18 h, the filter was washed to remove all the cells and blotted with anti-CPY (A6428, Thermo Fisher Scientific, Waltham, MA, USA). Antibodies were detected by enhanced chemiluminescence with ECL reagents (Amersham, Piscataway, NJ, USA).

For immunoprecipitation of HeLa extracts, cells were cultured in P100 culture dishes and cotransfected with 10 μg of pmCherry-ATG2A along with 10 μg of pEGFP-C3, pEGFP-WIPI4 or the indicated mutant derivatives. Twenty-four hours after transfection, cells were lysed and immunoprecipitation of GFP fusion proteins was performed with GFP-Trap beads (Chromotek, Planegg, Germany), following the instructions of manufacturer. Immunoprecipitated extracts were analyzed by 7.5% SDS/PAGE and immunoblotting with Living Colors DsRed (Clontech) and rabbit anti-GFP N-terminal (Sigma-Aldrich) antibodies. Antibody detection was performed as described above.

### Two-hybrid assays

*Saccharomyces cerevisiae* strain used for two-hybrid assays was Y187 or the isogenic derivative OVY546 (*Δatg18*) (Supplementary Material, Table S1). X-gal filter lift assays were performed as described previously (74) and developed for 2 h. For quantitative assays, eight independent transformants were grown to logarithmic phase in SD medium. β-galactosidase was assayed in permeabilized yeast cells and expressed in Miller units (75).

## Supplementary Material

Table_S1_ddab225
