# Supplementary material for: A conserved ATG2 binding site in WIPI4 and yeast Hsv2 is disrupted by mutations causing β-propeller protein-associated neurodegeneration
Source: Hum Mol Genet. 2021 Aug 9;31(1):111–21. doi: 10.1093/hmg/ddab225 (PMC8682751; doi:10.1093/hmg/ddab225)
Supplement: Table_S1_ddab225 [file table_s1_ddab225.doc]

**A conserved ATG2 binding site in WIPI4 and yeast Hsv2 is disrupted by mutations causing β-Propeller Protein-Associated Neurodegeneration**

Miranda Bueno-Arribas, Irene Blanca, Celia Cruz-Cuevas, Ricardo Escalante, María-Angeles Navas and Olivier Vincent

Supplementary data

**Table S1**. Yeast strains used in this study.

| Strain | Genotype | Reference |
| --- | --- | --- |
| Y187 | MAT**α** ura3-52 his3-200 ade2-101 trp1-901 leu2-112 gal4Δ, met15Δ, gal80Δ, URA3::GAL1UAS-GAL1TATA-lacZ, MEL1 | Clontech |
| YSL829 | BY4741; MAT**α** his3Δ1 *leu2Δ0 met15Δ0 ura3Δ0 VPS17-mCherry::KanMX6* | Gift S. Leon |
| Y00000 | BY4741; MAT**α** his3Δ1 *leu2Δ0 met15Δ0 ura3Δ0* | Euroscarf |
| OVY375 | BY4741; MAT**α** his3Δ1 *leu2Δ0 met15Δ0 ura3Δ0 vps13Δ::kanMX4* | Open Biosystem |
| OVY380 | BY4741; MAT**α** his3Δ1 *leu2Δ0 met15Δ0 ura3Δ0 hsv2Δ::kanMX4* | Open Biosystem |
| OVY384 | BY4741; MAT**α** his3Δ1 *leu2Δ0 met15Δ0 ura3Δ0 atg2Δ::kanMX4* | Open Biosystem |
| OVY499 | W303-1A; MAT**α** ura3-*1 his3-11,15 leu2-3,112 trp1-1 ade2-1 can1-100 hsv2Δ::natMX4 atg2Δ::hphNT1 his3::ATG2-3mcherry-HIS3* | This study |
| OVY503 | W303-1A; MAT**α** ura3-*1 his3-11,15 leu2-3,112 trp1-1 ade2-1 can1-100 atg2Δ::hphNT1 his3::ATG2-3mcherry-HIS3 VPS17-GFP::KanMX6* | This study |
| OVY510 | W303-1A; MAT**α** ura3*-1 his3-11,15 leu2-3,112 trp1-1 ade2-1 can1-100 atg2Δ::hphNT1 atg14Δ::natMX4 his3::ATG2-3mcherry-HIS3 VPS17-GFP::KanMX6* | This study |
| OVY528 | W303-1A; MAT**α** ura3*-1 his3-11,15 leu2-3,112 trp1-1 ade2-1 can1-100 hsv2Δ::natMX4 ura3::HSV2-GFP-URA3* | This study |
| OVY541 | W303-1A; MAT**α** ura3*-1 his3-11,15 leu2-3,112 trp1-1 ade2-1 can1-100 hsv2Δ::natMX4 atg2Δ::hphNT1 ura3::HSV2-GFP-URA3* | This study |
| OVY543 | W303-1A; MAT**α** ura3*-1 his3-11,15 leu2-3,112 trp1-1 ade2-1 can1-100 hsv2Δ::natMX4 atg2Δ::hphNT1 his3::ATG2-3mcherry-HIS3 VPS17-GFP::KanMX6* | This study |
| OVY545 | W303-1A; MAT**α** ura3*-1 his3-11,15 leu2-3,112 trp1-1 ade2-1 can1-100 hsv2Δ::natMX4 atg2Δ::hphNT1 his3::ATG2-3mcherry-HIS3 ura3::HSV2-GFP-URA3* | This study |
| OVY546 | MAT**α** ura3-52 his3-200 ade2-101 trp1-901 leu2-112 gal4Δ, met15Δ, gal80Δ, URA3::GAL1UAS-GAL1TATA-lacZ, MEL1, *atg18Δ::natMX4* | This study |
